# Supplementary figures and images for: Envelope reconstruction of speech and music highlights stronger tracking of speech at low frequencies
Source: PLoS Comput Biol. 2021 Sep 17;17(9):e1009358. doi: 10.1371/journal.pcbi.1009358 (PMC8480853; doi:10.1371/journal.pcbi.1009358)

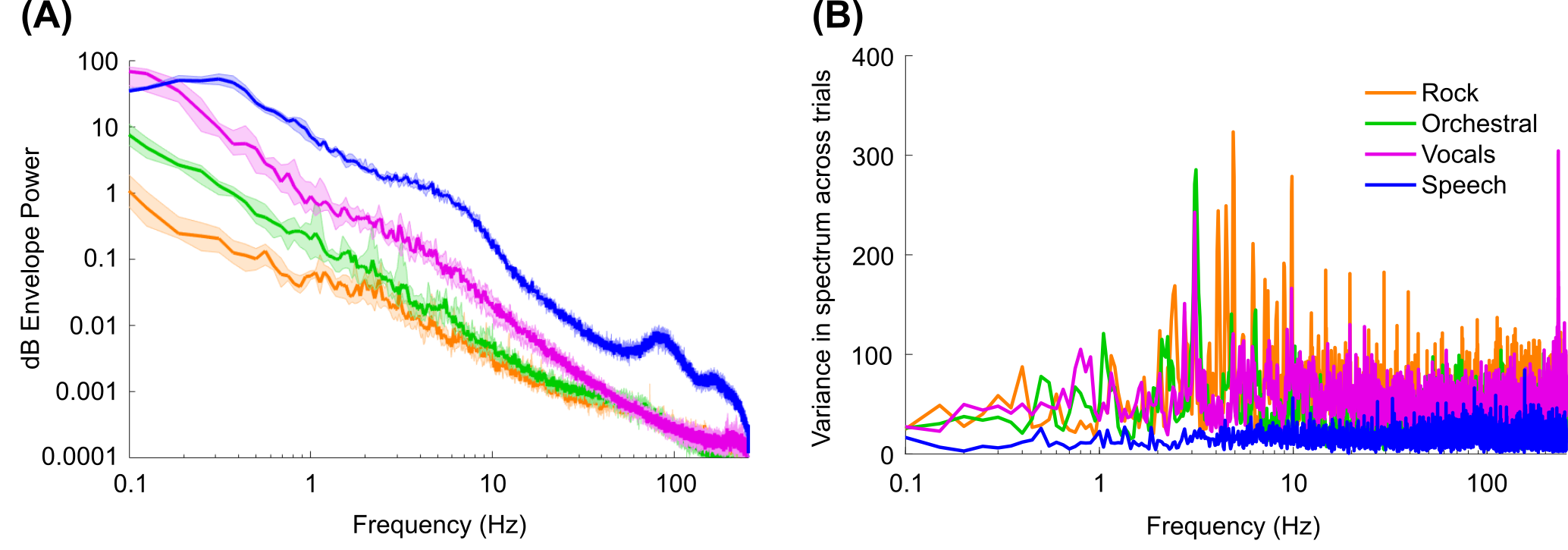

Supplement: S1 Fig — (A) Envelope power spectra for each stimulus type prior to normalizing by the EEG power spectrum, as in Fig 1B. Lines indicate the median across stimuli of each type, and shaded regions indicate 95% quantiles of the distribution of 1000 bootstrapped median values. (B) Variance in the spectrum across trials. Because the speech trials all come from a single audiobook with one talker, they are more spectrally-similar to each other than the music stimuli. (TIF) [file pcbi.1009358.s001.tif]

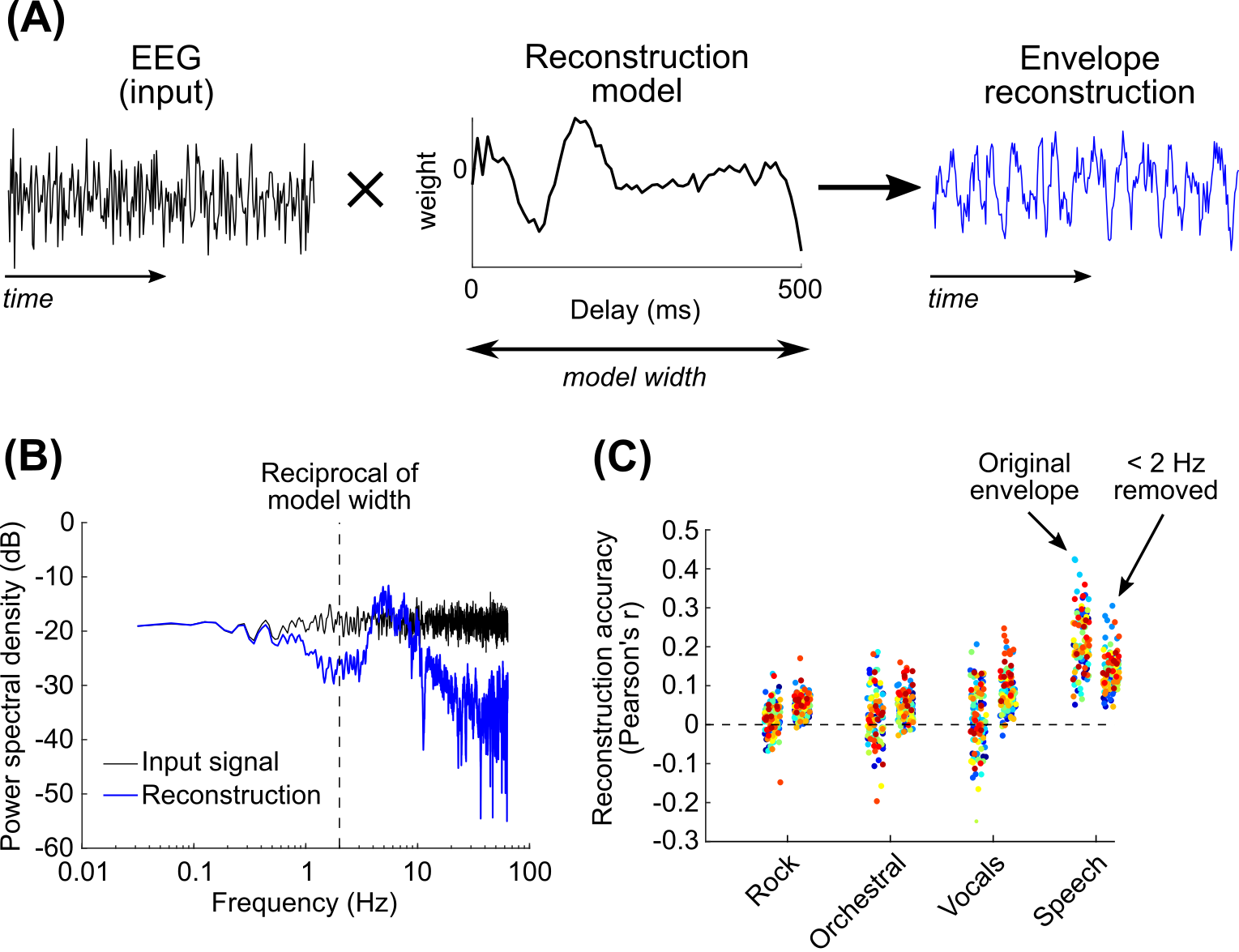

Supplement: S2 Fig — (A) Ridge regression was used to reconstruct the envelope from the EEG. Shown in the middle is the averaged reconstruction model across subjects and EEG channels for speech, where ridge regression was used to fit the model. Normally, the reconstruction model takes EEG as an input, but to simulate the spectral effects of the reconstruction model on the input, we have used broadband noise as the input in this example, which has a flat frequency spectrum. (B) When looking at the spectrum of the reconstruction with respect to a broadband noise input (black), it is clear that the reconstruction model accentuates certain frequencies and reduces higher frequencies (blue). However, it has no effect on the magnitude for frequencies corresponding to less than 2x the model width (1 Hz in this example), although it does add a delay that produces a phase shift at these frequencies (not shown). (C) The presence of low-frequency tracking has an effect on reconstruction accuracies. When the envelope and the EEG are highpass filtered by removing the moving average of the model width (500 ms), speech envelope reconstruction significantly drops, showing neural tracking at low frequencies untouched by the model. In contrast, reconstruction accuracies for the music stimuli significantly improves without these lower frequencies. Each color represents the testing reconstruction accuracies for one of the subjects. (TIF) [file pcbi.1009358.s002.tif]

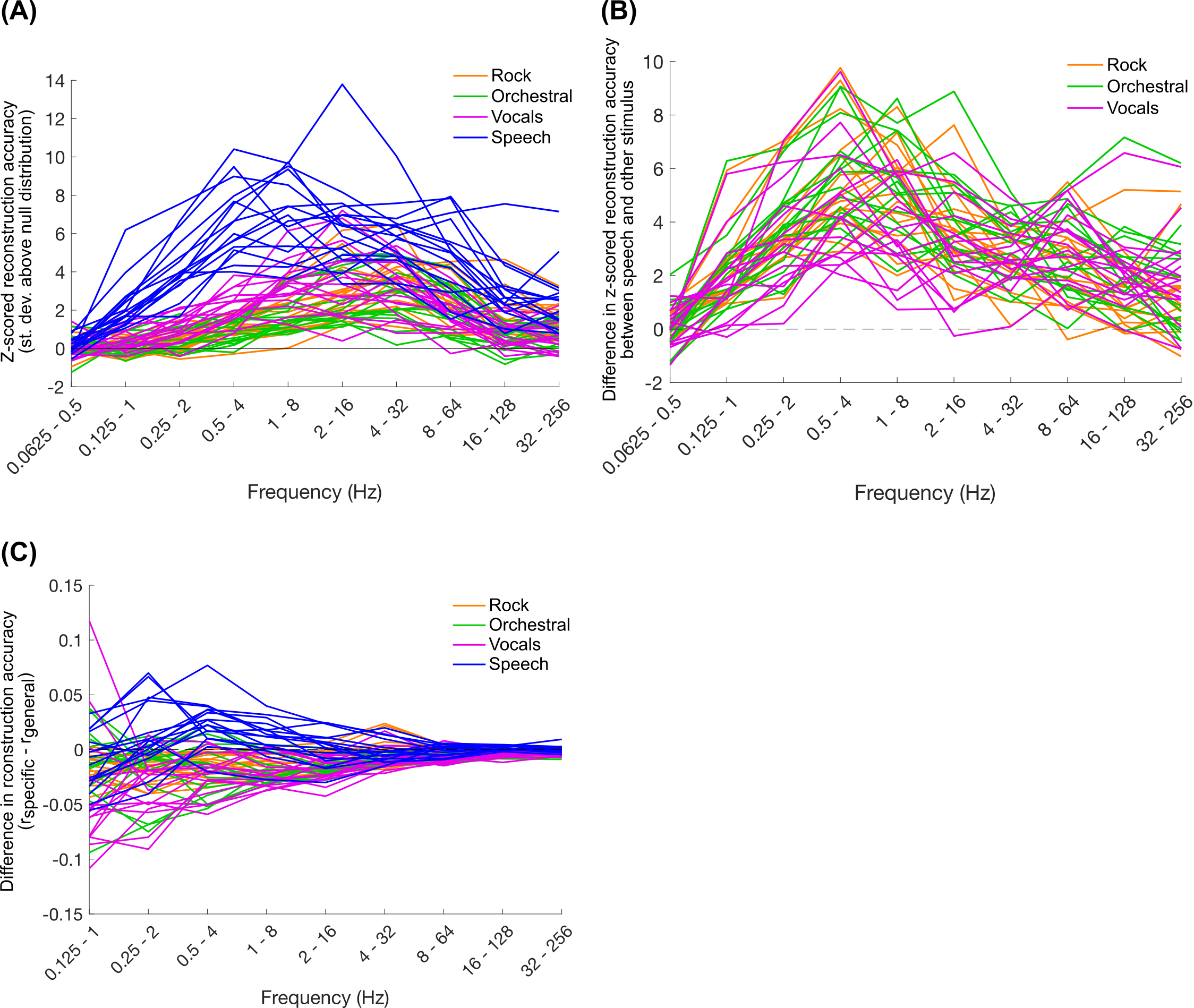

Supplement: S3 Fig — (A) Z-scored reconstruction accuracies for each stimulus type (compare to Fig 3D). (B) Difference between z-scored reconstruction accuracy for speech and each of the other stimulus types (compare to Fig 3E). (C) Difference between stimulus-specific and stimulus-general reconstruction accuracy (compare to Fig 5A). (TIF) [file pcbi.1009358.s003.tif]

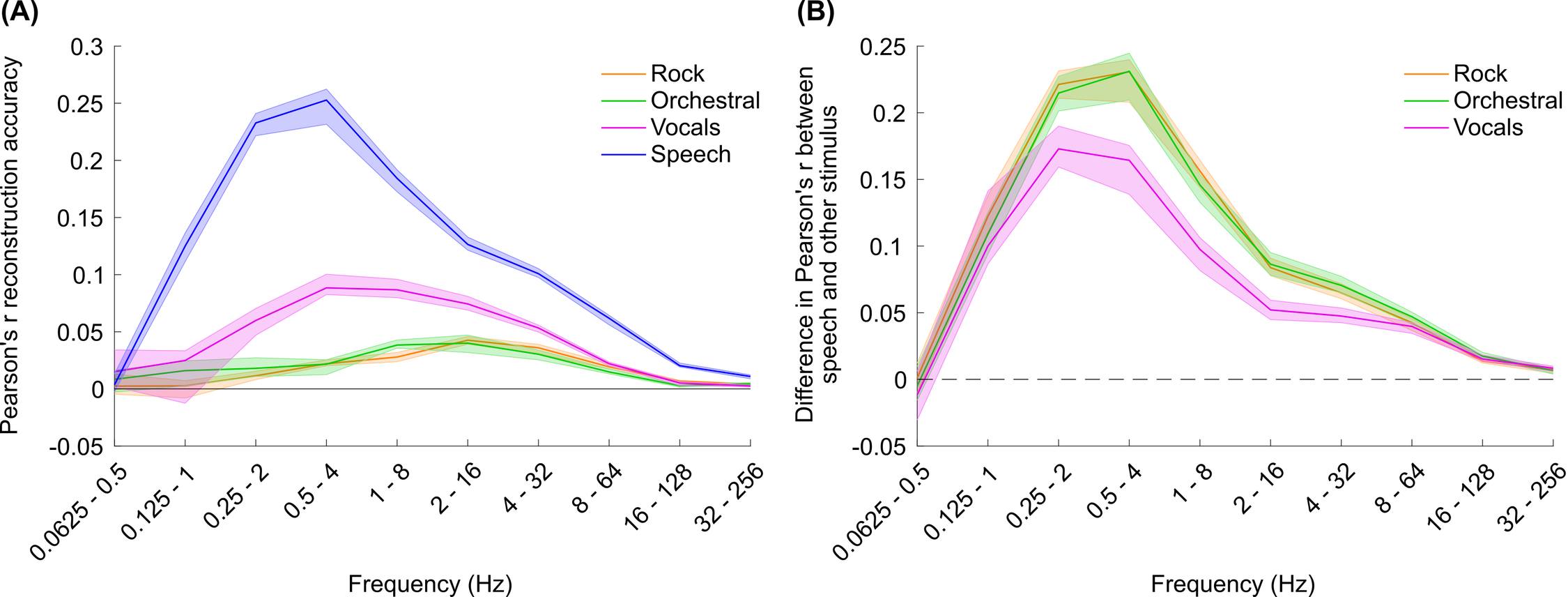

Supplement: S4 Fig — (A) Envelope reconstruction accuracy based on Pearson’s r, without the z-scoring used in the manuscript. (B) The difference in Pearson’s r between the speech reconstructions and the reconstructions for each stimulus type shown. These were plotted identically to Fig 3D and 3E, showing the median and 95% quantiles across trials and subjects. (TIF) [file pcbi.1009358.s004.tif]

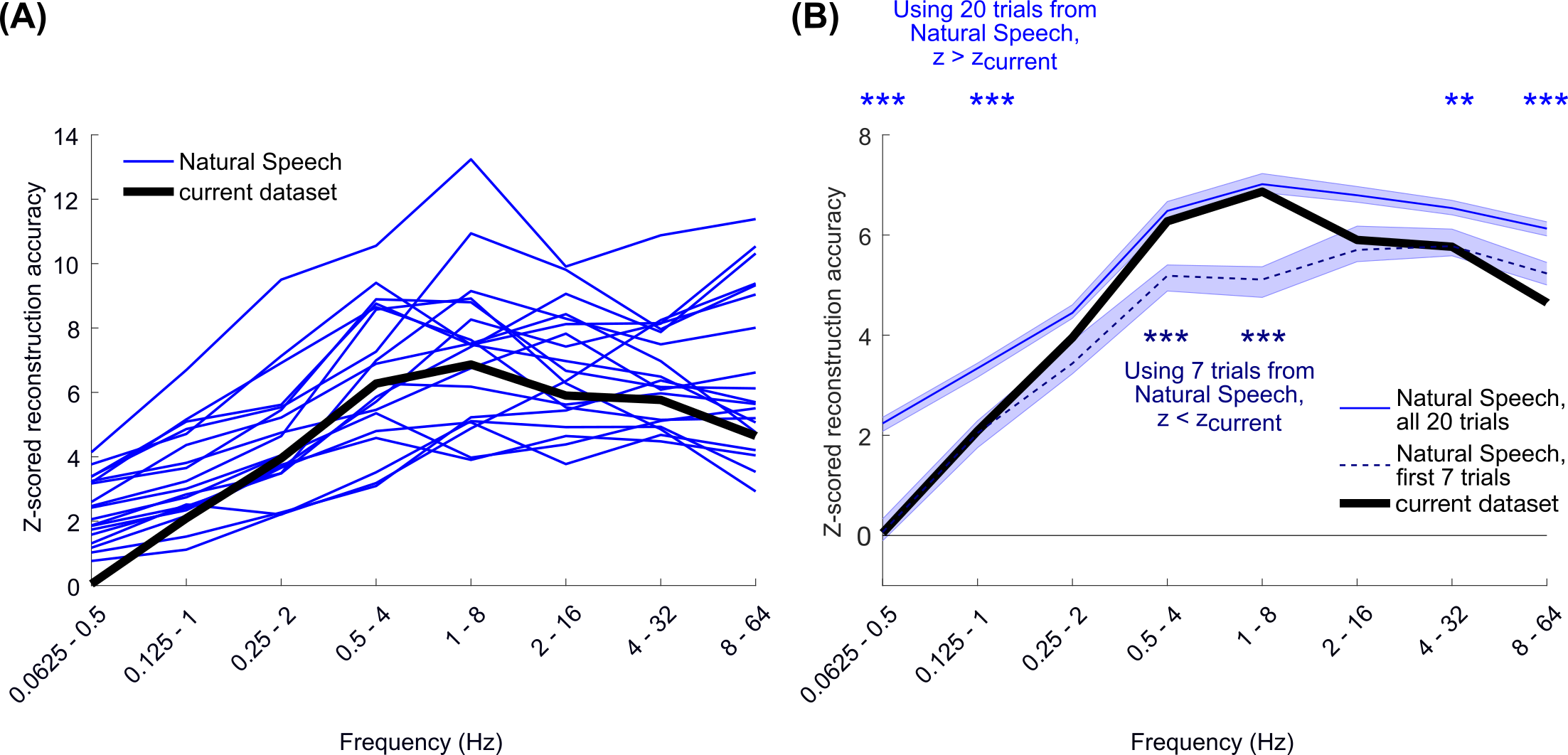

Supplement: S5 Fig — Note that the Natural Speech dataset contained 20 trials of the audiobook, whereas the current dataset in the study only contained the first 6–7 trials (7 for most subjects, see S1 Table). (A) Shown are the reconstruction accuracies for all 19 subjects in the Natural Speech dataset, averaged across 20 trials. (B) We looked at reconstruction accuracies using all 20 trials of Natural Speech (blue, same results as A) and only the first seven trials (darker blue, dashed line in B). Wilcoxon’s rank-sum test with Bonferroni correction for 16 comparisons was used to compare reconstruction accuracies between datasets; blue shows the comparisons with all 20 trials of Natural Speech, and darker blue shows comparisons is using just the first seven trials (** p < 0.01; *** p < 0.001). In both instances, reconstruction accuracies were comparable to the current dataset and higher than the reconstruction accuracies for the other stimuli (see Figs 3D and S1). Note, however, that using all 20 trials produces above-chance reconstruction accuracies for the lowest frequency model, 0.0625–0.5 Hz. The reconstruction accuracies drop to chance when only seven trials are used. This indicates that the chance performance we observed in the current dataset may not be due to a low-frequency limitation on neural tracking of the speech envelope and may instead be a result of the limited amount of data in this study. (TIF) [file pcbi.1009358.s005.tif]

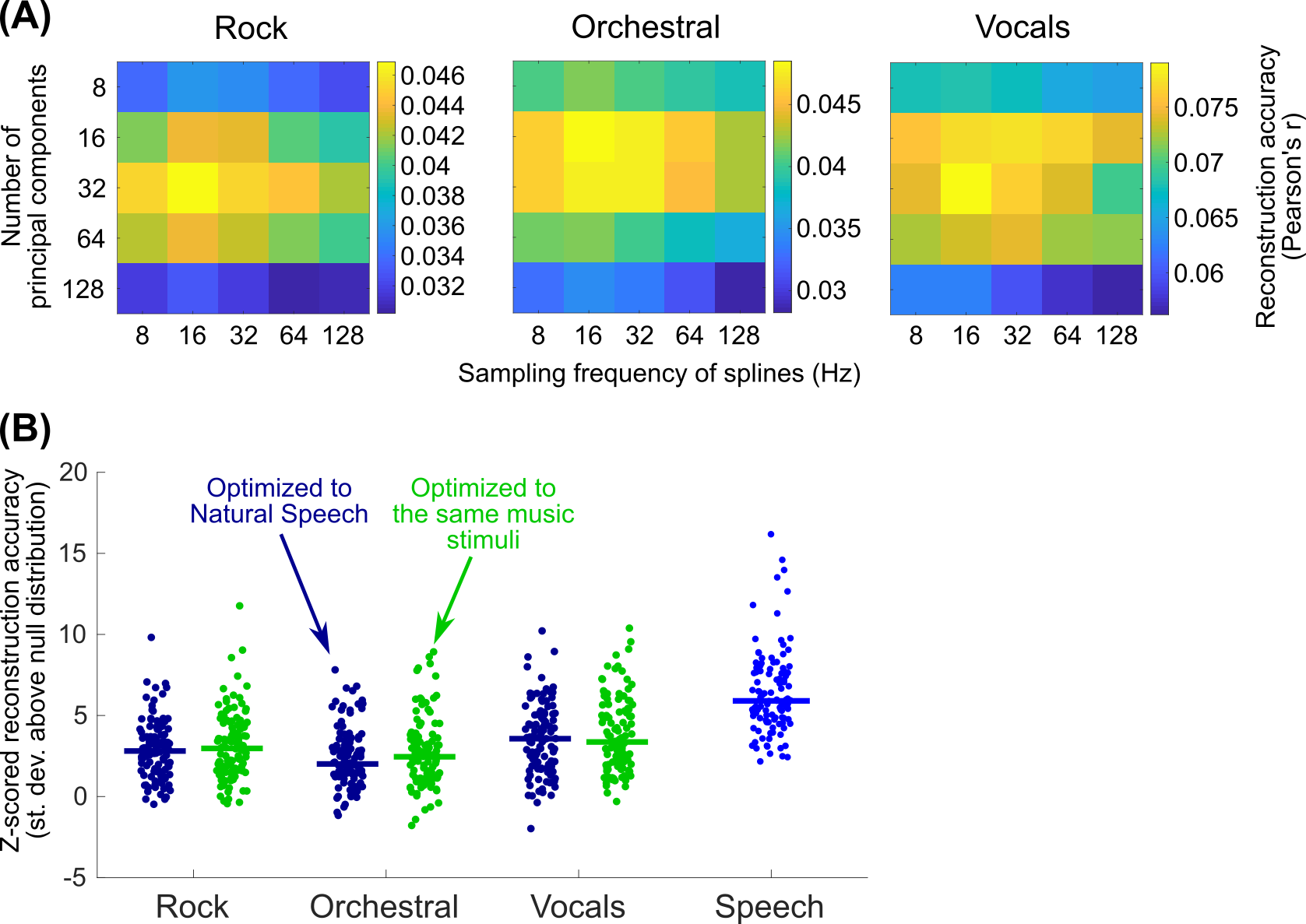

Supplement: S6 Fig — Here, we tested if music envelope reconstruction performs as well as speech if we optimize the hyperparameters for the music stimuli. (A) Using the same 500 ms model window as before, we found the optimal hyperparameter pairs for each stimulus type that maximized the average envelope reconstruction accuracy across subjects. These optimal hyperparameters were different than those found for Natural Speech (Rock = 16 Hz spline knots, 32 principal components (PCs); Orchestral = 16 Hz, 16 PCs; Vocals = 16 Hz, 32 PCs; Natural Speech = 32 Hz, 64 PCs). (B) We then computed the z-scored reconstruction accuracies (as in Fig 3) using these optimal hyperparameters. For each music stimulus, the dark blue dots on the left are the trial-by-trial reconstruction accuracies for all subjects using the Natural Speech hyperparameters (the same datapoints as those used to create Fig 3D), and the green dots on the right are using the music-optimized hyperparameters. The blue dots for the speech z-scored accuracies are based on the Natural Speech hyperparameters. Lines indicate the median values across trials and subjects. Even after optimizing the hyperparameters to the music stimuli, speech envelope reconstruction still outperforms music (Wilcoxon rank-sum relative to speech: zrock = 9.17, zorchestral = 9.84, zvocals = 7.16, p < 0.001 for all comparisons). (TIF) [file pcbi.1009358.s006.tif]

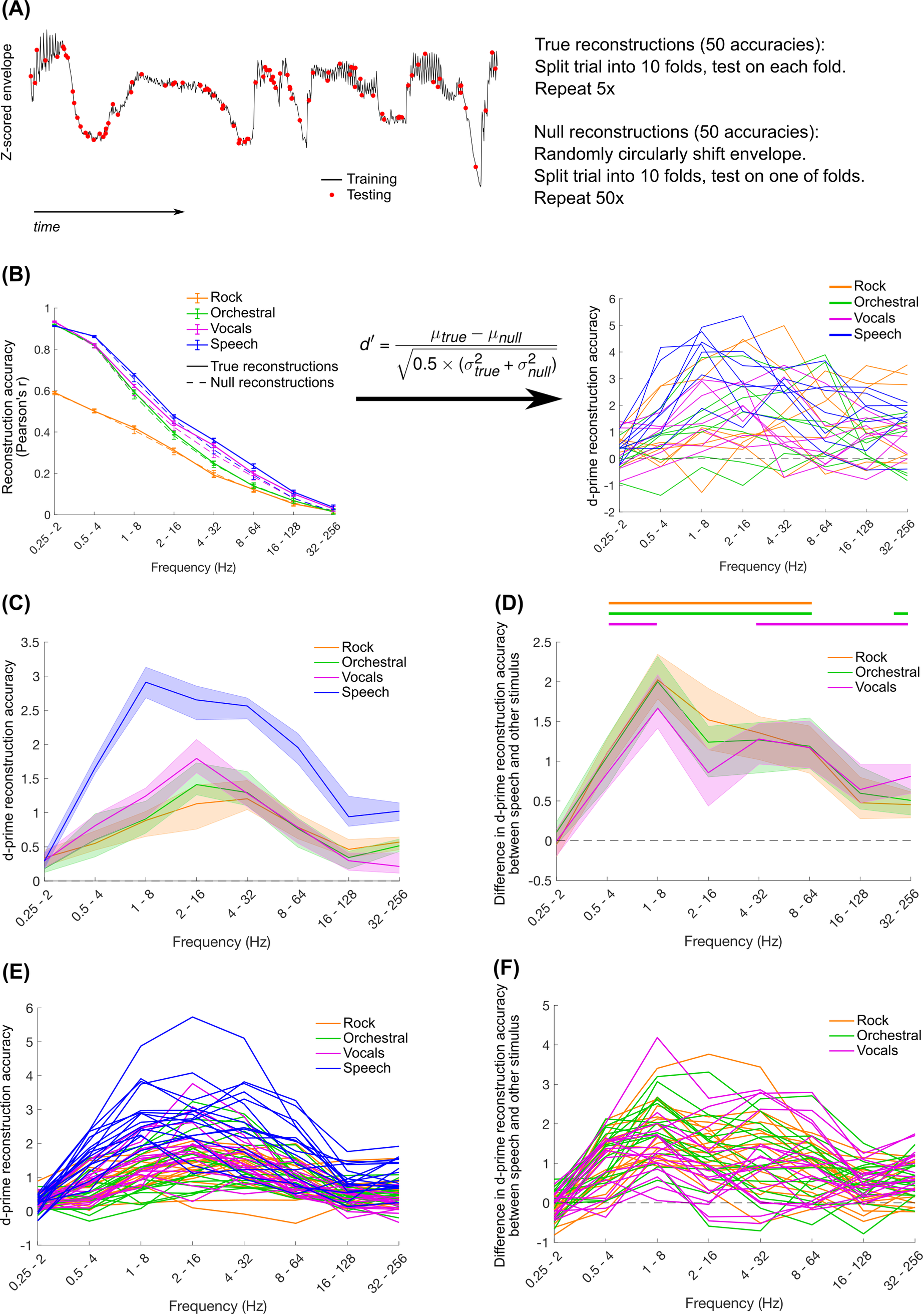

Supplement: S7 Fig — To control for this, we looked instead at within-trial reconstruction accuracy. (A) To get reconstruction accuracies for each trial, we split the trial into 10 evenly-sized folds, where each fold contained a random sampling of the data in the trial. This was done in order to maximize the consistency in the EEG covariance and envelope spectrum across folds. Then models were fit on all trials with one fold left out and tested on the left-out fold. This was repeated 5 times using a new random sampling of folds each time, giving a total of 50 reconstruction accuracies (Pearson’s r) for each trial. To get a null distribution of accuracies, the stimulus envelope was randomly circularly shifted, 1/10th of the data was randomly sampled for testing, and the rest of the data was used for training. This was repeated 50 times to get 50 null reconstruction accuracies. (B) Because testing data is highly correlated with training data using this method, both the true and null reconstruction accuracies increase as lower frequencies are used for modeling. To correct this, we computed a d-prime reconstruction accuracy based on the distribution of true and null reconstruction accuracies. (C, D) Firstly, d-prime reconstruction accuracies dropped to zero for the 0.25–2 Hz model. This is a consequence of the reduced amount of data available in each trial; using lower-frequency models (with larger model windows) generated warnings in Matlab indicative of overfitting. But that aside, across all frequency ranges, d-prime reconstruction accuracy was significantly larger than all other music stimuli. Thick lines in D show significance of a permutation test comparing speech d-prime to each of the different stimulus types, p < 0.001 with Bonferroni correction for 24 comparisons. Plots (E and F) show the same results as C and D, respectively, for individual subjects. Overall, this indicates that, even when doing within-trial reconstructions to avoid the effects of cross-trial variance, speech is [file pcbi.1009358.s007.tif]

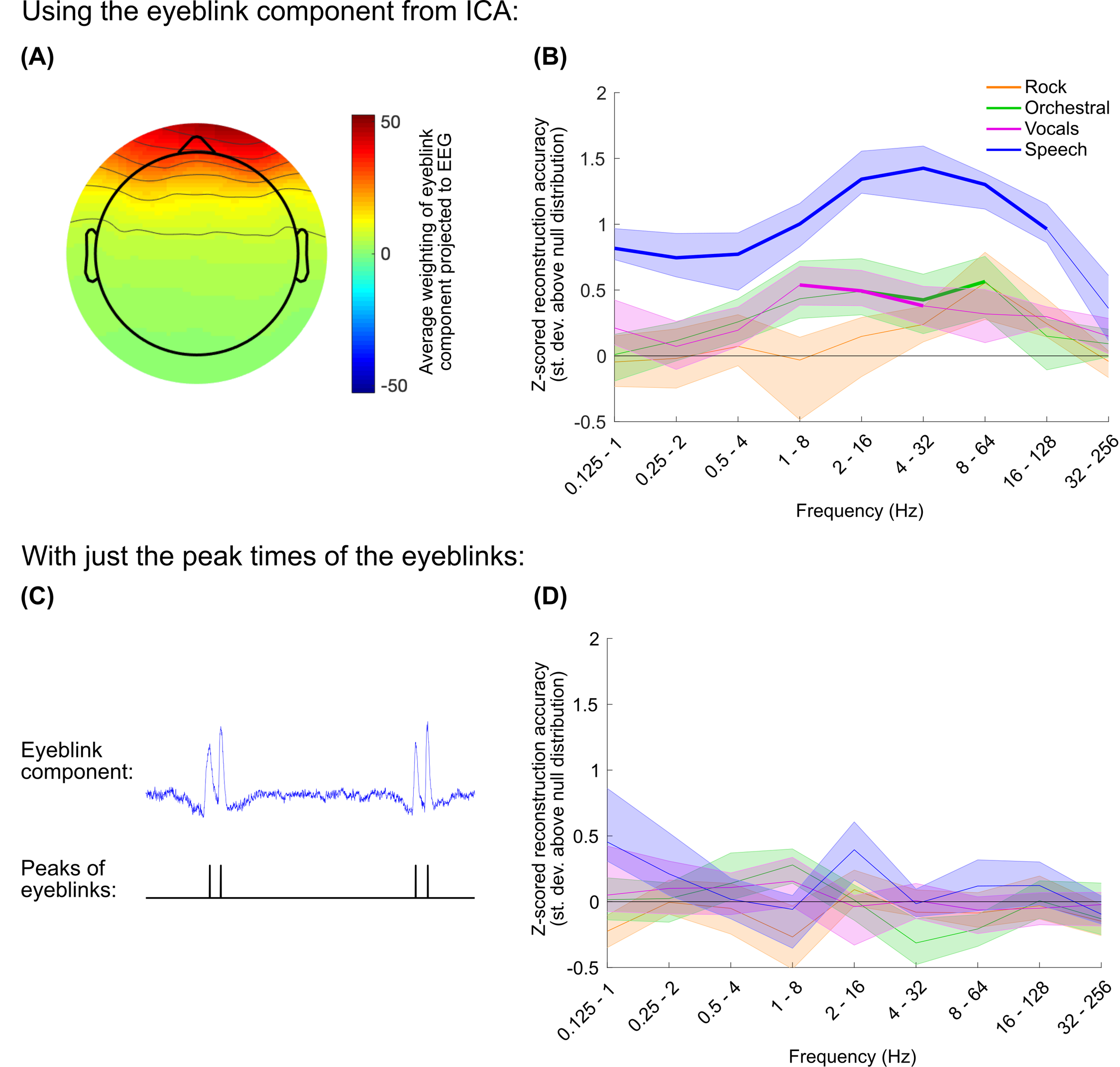

Supplement: S8 Fig — First, the EEG was highpass filtered by removing a moving average window 16 s long. Then the eyeblink component was calculated for each subject using independent components analysis (ICA; specifically, fastICA, as in [30]) and then identified empirically by the topography of the projection weights (transforming from the independent component to EEG space) and the time course of the EEG signal. One subject was left out because we could not reliably get a single component of eyeblinks using ICA. (A) Shown are the projection weights for this component averaged across the other 15 subjects. (B) Reconstruction accuracies using the eyeblink component. Thick lines indicate values significantly larger than zero based on a Wilcoxon signed-rank test with Bonferroni correction for 36 comparisons (p < 0.001). After repeating the envelope reconstruction analysis, we found that the reconstruction accuracies for all stimuli are still above chance at higher modulation frequencies, but considerably smaller than before (compare to Fig 3D). Similarly, the speech reconstructions were still significantly better than music. While this could indicate the involvement of eyeblinks, it is also plausible that the eyeblink component contains residual neural activity that tracks the envelope, since the topography for the eyeblink component overlaps the spatial weightings of the envelope reconstruction models (Fig 4). Thus, we constrained the analysis further by creating another input signal from the eyeblink component that only contained onsets at the peaks of the eyeblinks. (C) For each subject, the eyeblink component was highpass filtered again at 1 Hz by removing the moving average of a 1 s window, and then an eyeblink trigger was set individually for each subject to automatically identify eyeblinks by threshold crossing. The peak times of the eyeblinks were identified and the peak onset vector was used as input for envelope reconstruction. This ensured the envelope reconstruction would only [file pcbi.1009358.s008.tif]

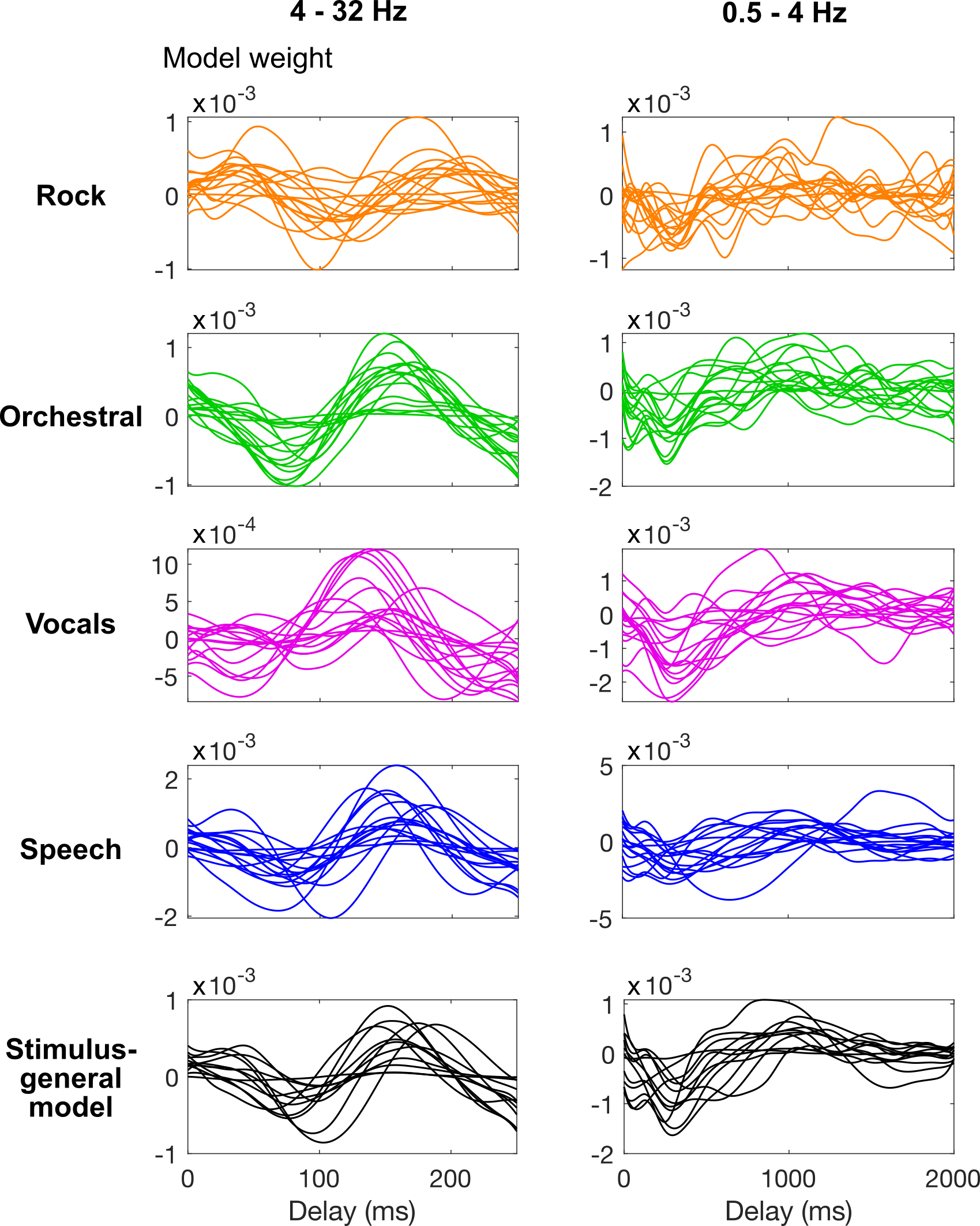

Supplement: S9 Fig — The models were converted from basis splines to delays, and then from principal components to EEG channels. The weights shown here were averaged across all 128 EEG channels (compare to Figs 4 and 5B). (TIF) [file pcbi.1009358.s009.tif]

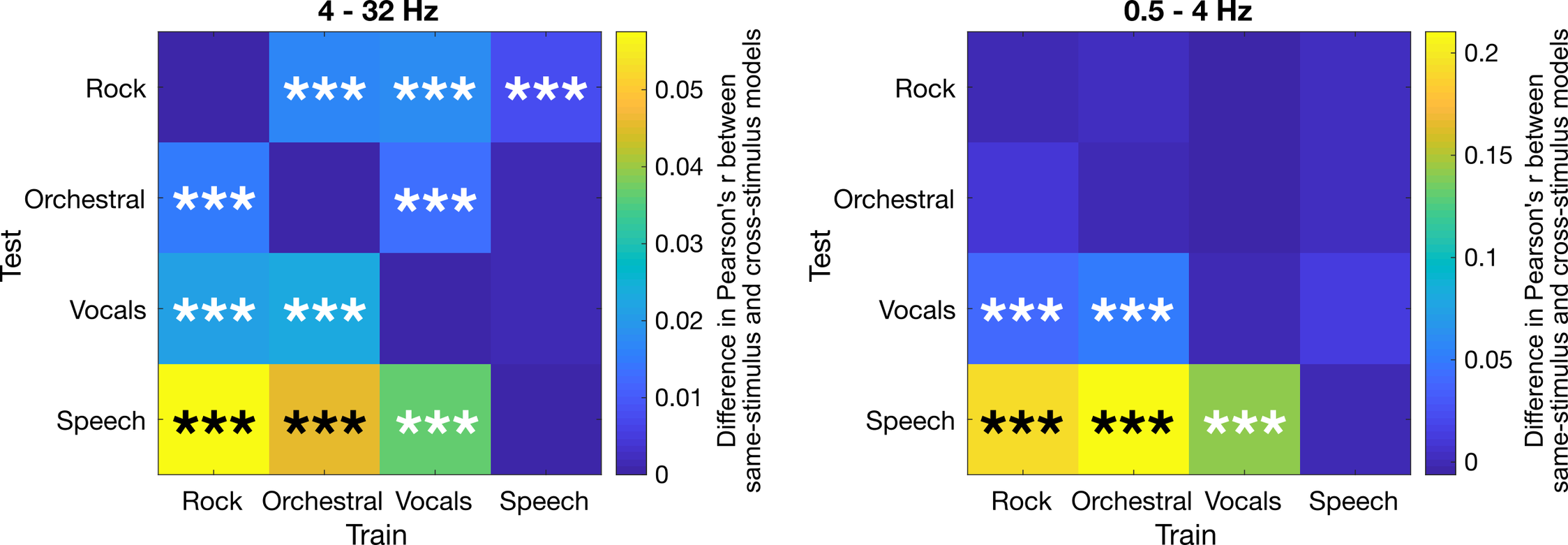

Supplement: S10 Fig — The “same-stimulus” model was trained and tested on the same stimulus type (this is identical to the “stimulus-specific” models from Fig 5 in the manuscript). The “cross-stimulus” models were trained on all of the presented stimuli for one stimulus type (the “Train” stimulus) and tested on each trial of another stimulus type (the “Test” stimulus). Shown here is the difference between the same-stimulus model and the cross-stimulus model. For example, the bottom left corner is the difference in reconstruction accuracy between a speech model trained on speech (same-stimulus) and a model trained on rock (cross-stimulus). Values greater than zero imply that the same-stimulus model outperforms the cross-stimulus model. Three asterisks (both white and black) indicate a significance of p < 0.001 for a Wilcoxon signed-rank test relative to a median value of zero after Bonferroni correction for 24 comparisons (this excludes the diagonals of each plot). (TIF) [file pcbi.1009358.s010.tif]

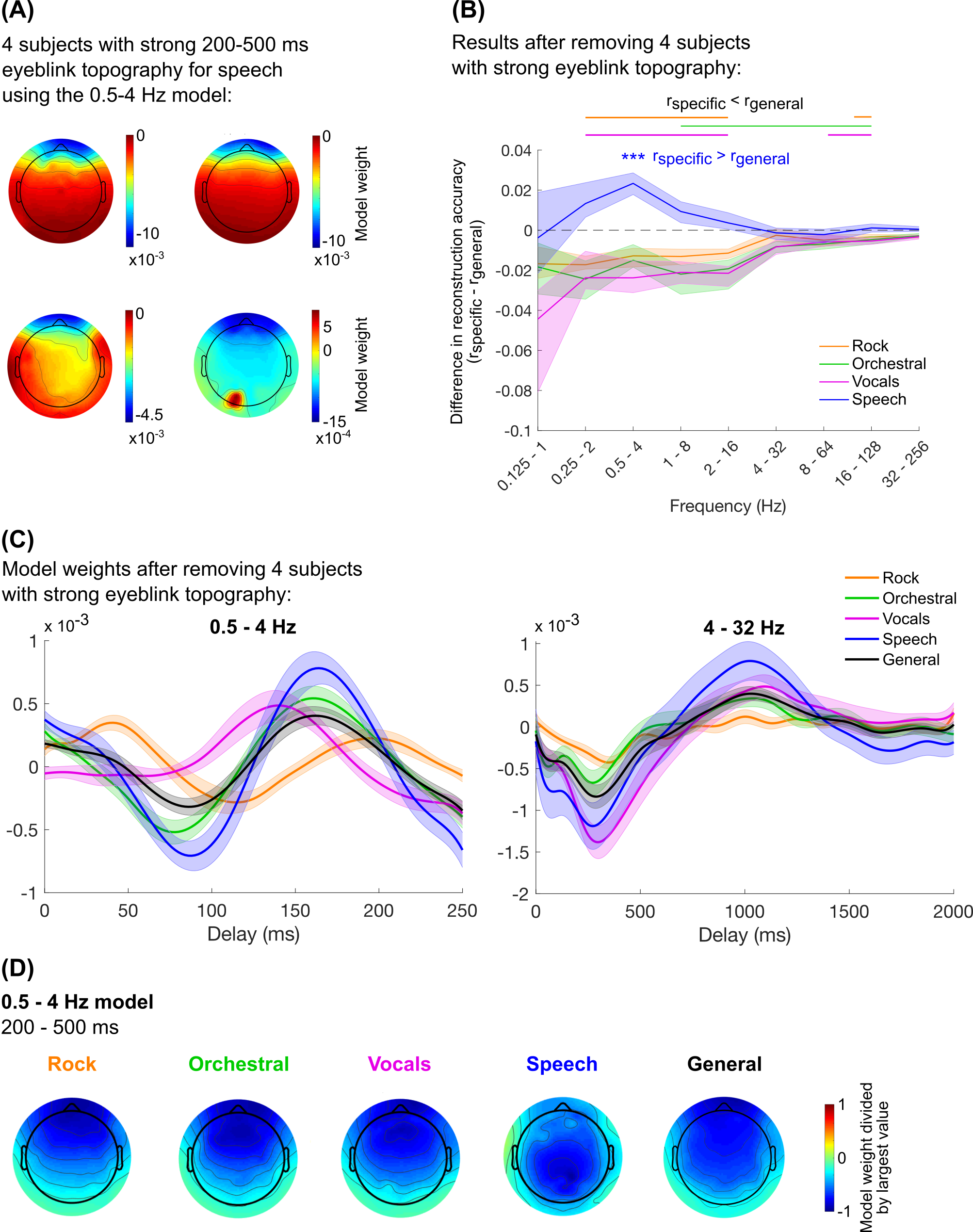

Supplement: S11 Fig — While, to our knowledge, no eyeblink-based speech envelope reconstruction has been reported in the past, a 300–400 ms frontal negativity is indicative of eyeblink contamination in evoked response analyses [42]. (A) We examined the topography of the weights between 200–500 ms for each individual subject and found four subjects with topographies strongly indicative of eyeblinks. (B) After removing these subjects from analysis, however, the stimulus-specific model for speech still outperformed the stimulus general model for 0.5–4 Hz (Wilcoxon signed-rank test with Bonferroni correction for 32 comparisons, p < 0.001), but this was no longer true for the 1–8 Hz model. (C and D) Additionally, the time course and topographies of the model weights were very similar to what was observed in our analysis using all 16 subjects (compare to Figs 4 and 5). (TIF) [file pcbi.1009358.s011.tif]

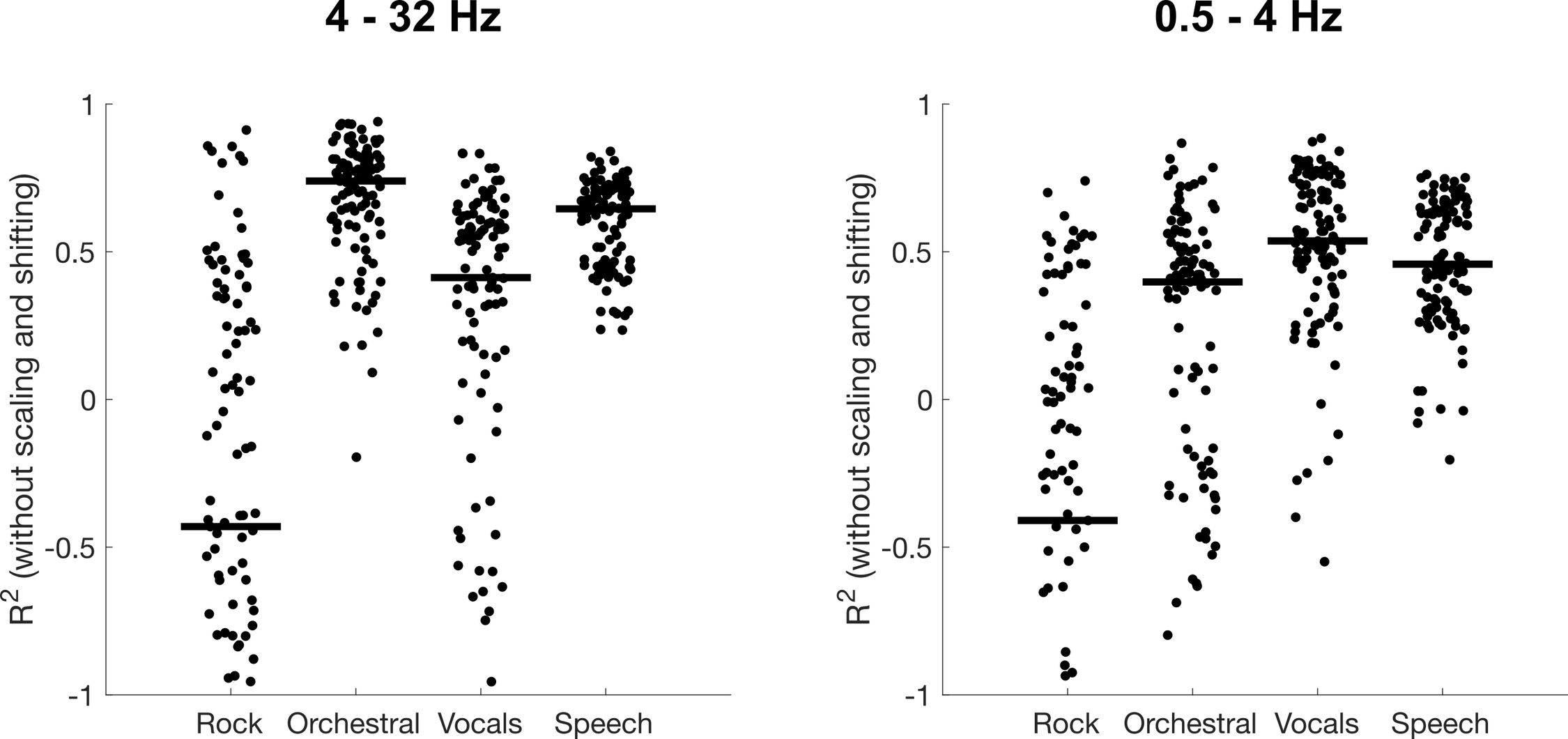

Supplement: S12 Fig — As in Fig 5E, this is shown for the 4–32 Hz and 0.5–4 Hz models. The y-axis has been restricted to a range from -1 to 1 for easier comparison of the medians (lines); datapoints below R2 are not shown. (TIF) [file pcbi.1009358.s012.tif]

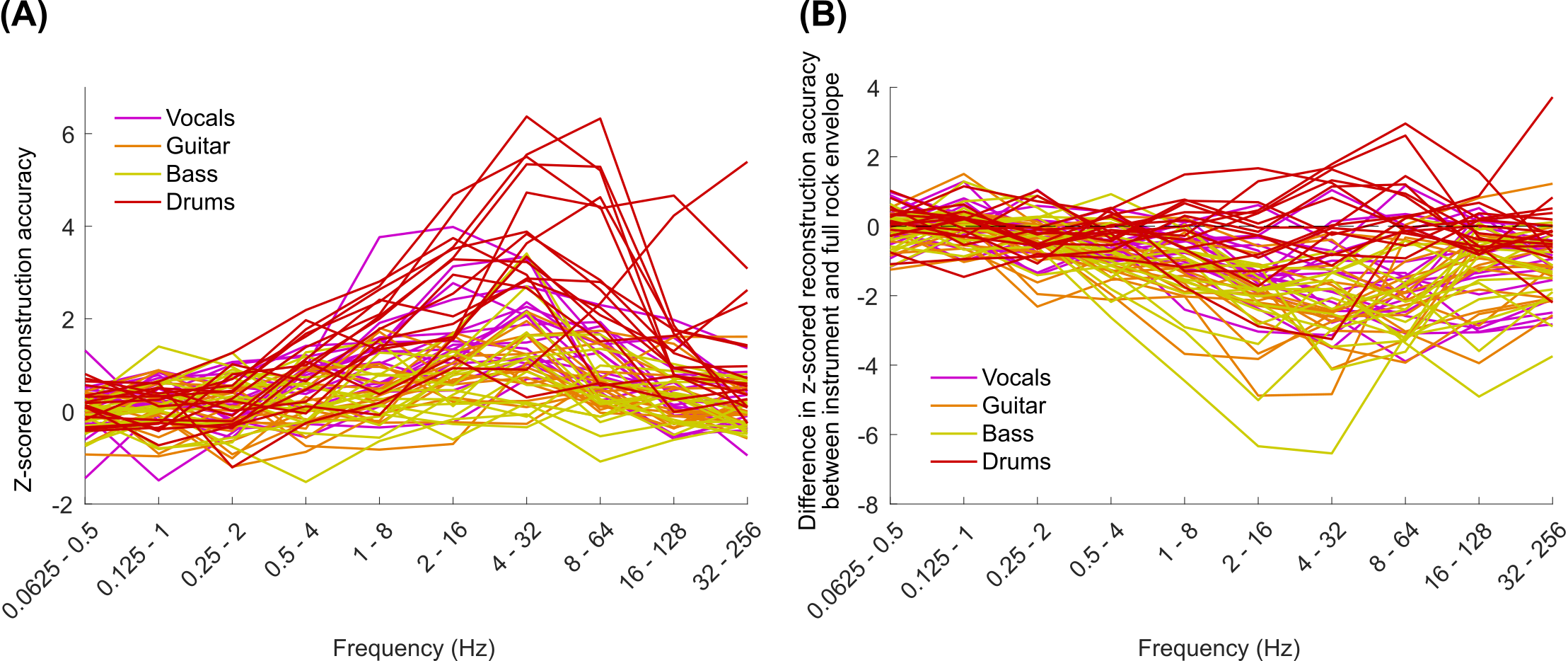

Supplement: S13 Fig — (A) and (B) are plotted identically to Fig 6A and 6B respectively. (TIF) [file pcbi.1009358.s013.tif]
