# Supplementary material for: Envelope reconstruction of speech and music highlights stronger tracking of speech at low frequencies
Source: PLoS Comput Biol. 2021 Sep 17;17(9):e1009358. doi: 10.1371/journal.pcbi.1009358 (PMC8480853; doi:10.1371/journal.pcbi.1009358)
Supplement: Attachment — Submitted filename: NeuroMusic_paper_v5p2_responses.docx [file pcbi.1009358.s015.docx]

**Envelope reconstruction of speech and music highlights stronger tracking of speech at low frequencies**

Responses to reviewer comments

We would like to thank the reviewers for their thorough and thoughtful comments on our manuscript. In these responses, we refer to the page numbers and line numbers in the marked version of the manuscript.

Note also that, following our resubmission of the manuscript, we were requested to include the Author Summary for the study. We have included this only in the clean version of the manuscript in order to prevent changing the line numbering of the marked version.

**Reviewer #1**:

*The authors explored whether the human brain tracks speech and music differently using EEG. Using a novel method that enabled them to examine envelope tracking in narrow frequency bands, they reported that speech was better tracked at all the frequency range they examined. Furthermore they found a different topography in weightings for speech-specific model reconstruction compared to other stimulus-general or stimulus-specific model reconstruction at the low frequency range (0.5-4 Hz). Overall, the MS is very well written and clear; the methods are sound.*

Thanks!

*I do not have specific major concerns but I would like the authors to be clearer on the following points:

1. The very low frequency range is particularly interesting but there are a few gaps in the MS in my mind. How good is the BioSemi EEG system sampling at very low frequency (~0.1 Hz)? What is hard to ascertain is that whether the noise floor is too big (either because of the equipment constraints or not having enough trials, c.f. Fig. S3) to conclude that tracking speech is better at "all" frequencies.*

The reviewer makes an interesting point about the limits of recording at a low frequency range. Here, however, we think the lower limit for speech tracking we observed was imposed by a limitation in the amount of data rather than caused by an increased noise floor. The Natural Speech dataset was collected using the same BioSemi system, and when we include more trials (28 instead of 7) the reconstruction accuracy at the lowest frequency range we measured was significantly above chance. We were expecting to see speech outperform music in a narrower set of frequencies, but with our analysis pipeline and stimuli we found speech outperform at all frequencies we analyzed. We have mentioned this in the paper: “Note that the Natural Speech dataset was collected using the same Biosemi system as the current dataset, so the limitation we observed here is unlikely to be an issue with the low-frequency noise floor of the EEG collection.” (l. 215-217)

We also agree that the reconstructions for music could be improved with more trials. In Figure 5 we show that the music reconstructions are worse with the stimulus-specific model than the stimulus-general model, which we think is either because of the difference in the amount of training data (5-6 trials compared to 20-23 trials respectively) or because the music-based models are simply worse at reconstructing the envelope (Figure S9 suggests that it is in part a data limitation issue). However, note that the difference in reconstruction accuracy between the two models is on average around 0.02-0.03, which is not enough to match the reconstruction accuracies for speech. For a better comparison to the stimulus-general to stimulus-specific analysis, I have included a plot of the median Pearson’s r (instead of z-scored r-values) for each stimulus type and its difference from speech (Figure S11). On average the difference is at least 0.05, which is still larger than the amount of improvement provided by the stimulus-general model.

*The analysis of the drum track is an interesting analysis to examine low frequency envelope tracking. But it is also debatable one would track every beat of the drum (during passive or even active listening). Rather, one suspects that you would care mostly the "strong" beat of each measure and acoustically, the relative magnitude would also be different on this beat. This is significant because instead of looking at the harmonics of these beats, one would argue the sub-harmonics would be more important. Thus for three songs mentioned in ll 282-283 with beats being below 2 Hz (i.e., less than 120 beats per minute; bpm), one could argue that listeners would be following at 1/4 of the rate (if there are 4 beats to a measure, i.e., 0.5 Hz). If the music is in a slower pace, it can greatly reduce to 0.1-0.2 Hz range.*

The reviewer also suggests that subjects may be perceptually tracking lower frequency fluctuations in the music stimuli, and suggests showing the reconstruction spectra at lower frequencies. We are familiar with previous research showing neural tracking at lower frequencies associated with metrical accents or phrases in music (Nozaradan et al, 2011; Baltzell et al, 2020). However, we think our analysis based on the amplitude envelope fails to capture that tracking, though it might be present. If there was significant tracking at these lower frequencies, we would expect the reconstruction accuracies to be above chance at the lower frequency range. At the higher frequency ranges, the EEG is filtered and examining reconstructions at those lower frequencies will not be informative. That said, we have included the following in the discussion to address this caveat: “Other studies have found significant neural tracking at the meter of the music (Nozaradan et al, 2011; Baltzell et al, 2020), and it is possible that our approach of examining envelope reconstruction with linear modeling fails to capture neural tracking at this frequency range, especially if it is only weakly present in the original envelope.” (l. 556-559)

*Thus, it would be useful to keep the frequency axis to show up to ~0.1 Hz range, e.g., Fig. 6C-E and Fig. S5 so that one could claim that speech tracking is much better than music for "all frequencies." If the very low frequencies are not conclusive, it's better to leave that as a caveat to be investigated in future studies.*

We appreciate the reviewer’s suggestion of restricting our x-axis to a minimum of 0.1 Hz, but we would prefer not to change those plots. In truth, we thought the model encompassing 0.0625 – 0.5 Hz was the lower limit for neural tracking of speech. Only after following up using a dataset with more trials did we find that accuracy was potentially above significance at this range. For transparency, we would prefer to keep these plots as is with an explanation of our follow-up analyses with the Natural Speech dataset.

*2. The parietal weighting difference in the speech-specific reconstruction is interesting, but the reader is left to wonder the greater significance of this besides that it is different from the stimulus-general reconstruction models and it is different from other stimuli. Are there any reasons why this difference would be parietal of origin?*

Because we did not do source localization analysis originally, we chose not to speculate too much on the origins of this signal. We note, however, that this topography has been found in other studies of speech processing. For example, it is the topography that is produced by neural tracking of semantic features (Broderick et al, 2018), and notably it is less present in non-native speakers (Di Liberto et al, 2020). Similar weightings have been found for speech tracking of phoneme and word surprisal as well (Gillis et al, 2021). Taken with these other lines of evidence, it suggests that this parietal topography indicates some process relevant for processing speech or other sounds with similarly low-frequency acoustic envelope fluctuations (though not the music in our dataset). Based on the topography and the use of mastoid references, we think this could be indicating activity from posterior temporal lobe, which is involved in processing speech (Hickok & Poeppel, 2007) but not music (Chen et al, 2021). However, we did not do source analysis, so we cannot say that with certainty.

We have included this in the discussion: “Recent studies also found that parietal weighting was increased for tracking phoneme and word surprisal (Gillis et al, 2021) as well as semantic tracking of speech for native speakers but not non-native speakers (Di Liberto et al, 2021). The parietal weighting could be indicative of language-specific processing in the posterior temporal lobe (Hickok & Poeppel, 2007) which was recently shown to be absent when listening to music (Chen et al, 2021), but because the activations are broad and without source localization it is difficult to identify the location definitively.” (l. 472-478)

*3. Since this paper would be of interest to researchers that are interested in music processing, it would be nice to have the musical terms to be more precise. The term "classical" is loaded. I think how it is used in this MS is the colloquial reference of instrumental music (that's of the Western Tonal Tradition) as well as it not being contemporary. Table S1 suggests that there are actually film music included and it's contemporary. I think using the term "orchestral / instrumental music" would be more appropriate. (It depends on whether the piano version or the orchestral version of Claire de Lune was used and if it's not piano, you can drop "instrumental" all together).*

All instances referring to the “classical” stimuli have been changed to “orchestral”.

*Minor comments:
Ln 100: rephrase "we expected that music may track speech comparably". You probably meant "music enveloping tracking would be comparably to that of speech" or to that effect.*

We changed this to: “we expected that music envelope tracking would be comparable to speech…” (l. 105-106)

*Fig. 5F: circular shift plots: What are the units for the vertical axis (cycle?) and can one contextualize what those values mean in a circular shift? Perhaps more details to guide the readers would be beneficial.*

We added a sentence briefly clarifying how the circular shift plot relates to the model weights: “Additionally, the circular shift of the stimulus-general model matched the expected shift from the averaged model for vocals (Figure 5f), showing a slight negative shift that was equal to amount of lead in the peaks of the vocals model relative to the stimulus general model”. (l. 326-329)

**Reviewer #2**:

*This study compares envelope coding in EEG activity during passive listening to speech and music. The authors use a novel stimulus reconstruction approach that band-limits each model to operate within a 3-octave range in the neural data, with separate models trained and tested across different bands that range from phrase-level modulations (<0.5Hz) to putative pitch-tracking (>32Hz). Based on prior work, the authors hypothesize that envelope tracking across music and speech may be comparable at higher modulation frequencies above 2Hz. The notable differences in modulation spectra that exist between the different stimulus categories are acknowledged, and the null hypothesis is that relative reconstruction accuracies across categories directly reflect these stimulus differences. Using within-category models (trained and tested on each stimulus independently) the results are broadly consistent with the null hypothesis, whereby speech reconstruction outperforms music across all frequencies. Slight differences are found in the modulation frequency at which peak accuracy occurs (lower for speech than music). To disentangle general-purpose auditory coding from domain-specific mechanisms, models are then trained/tested using data pooled across stimuli and compared with the performance of stimulus-specific models. While general vs. specific models yield similar performance at higher frequencies, speech-specific models are found to outperform the general models at lower frequencies (0.5-4Hz), which is driven by a subset of parietal electrodes. The authors use this finding to claim that “below 1Hz mechanisms exist that specifically track speech”.

On the whole I enjoyed reading this paper. The manuscript is well written, the data seems appropriately collected, analyses are mostly effective in testing the hypothesis, and statistical tests are appropriate.*

Thanks!

*However, given the nature of the differences in modulation spectra between stimuli, I have major concerns with the ultimate claim that low-frequency envelope tracking is specific to speech (see below). Because of this concern, the novelty of the current study is largely methodological rather than theoretical. It is my opinion that publication in PLoS Comp Bio is warranted if the authors significantly dial back their claims of speech-specificity and address the specific issues listed below.*

We thank the reviewer for highlighting this concern, and we agree that there are many caveats to this study which could weaken our argument for speech-specificity. In the previous draft, our intention was to highlight the presence of some channel weightings that was present “uniquely” for speech in comparison with our other stimuli. However, we tried to address some of those concerns in the discussion, suggesting other ways the uniqueness could be validated further.

In the most recent version, we have also made additional changes to dial back on the claims of speech-specificity, as the reviewer suggested. Specifically, we changed the title of the manuscript to “…stronger tracking of speech…”, instead of “…unique tracking of speech…”. We have also changed “uniquely” to “stronger” more “more strongly” throughout the manuscript.

*Major:

My main concern relates to the degree to which envelopes of speech differ from those in each music category. Figure 1A shows examples of slow modulations in speech (presumably sentence/phrase structure that results in the 0.5Hz peak). This modulation structure is virtually absent in the two instrumental categories, and only minimally present in vocals. This all seems intuitive - a rock song isn’t likely to be interspersed with silences in the same way an audiobook is.

This is problematic in two ways:

1. Firstly, at these very low modulation rates, if the signal is coming primarily from speech and not music - is it really surprising that the speech-specific model outperforms the stimulus-general model (the latter would essentially just have added noise due to low SNR of music)? This could also be the case if the neural response to power in a given modulation band wasn't linear across the range of stimulus-space occupied by both music and speech.*

We agree that the result that speech does better at low frequencies is not surprising on its own, and it is in agreement with the null hypothesis. However, if the reason speech does better was due to a difference in SNR, we would not expect the performance to differ from the stimulus-general model because model performance is based on correlations (smaller weights would produce a smaller reconstruction that is still equally correlated with the envelope). As long as the spatiotemporal responses are similar across stimulus conditions, we would expect the reconstruction to be similarly correlated to the original. The fact that it is not indicates a difference in the spatiotemporal response (particularly the spatial response) and not just SNR.

*2. Secondly (and perhaps more critically), the current study assumes that a fixed encoding mechanism exists across all frequencies. However, there may be two distinct mechanisms taking place for encoding envelope modulations at low and high frequencies respectively. This is what is suggested by ECoG research (e.g. see Hamilton et al., 2018) in which distinct neural populations in the STG respond to phrase-level onsets, independent from populations that encode the faster modulations in the continuous stream. Ultimately then, there may exist two different envelope mechanisms that are both domain-general. However, since the low-frequency structure only exists for speech within the current stimuli, encoding of slow envelope modulations are simply masquerading as speech-specialized.*

We agree with the reviewer that there are (at least) two different mechanisms involved in tracking low and high frequencies respectively. By analyzing the different frequency bands of envelope tracking individually, we were hoping to isolate the different spatiotemporal responses corresponding to low and high frequency tracking. Our comparison of stimulus-specific vs stimulus-general model performance was intended to address the reviewer’s second concern: that the low- and high-frequency mechanisms are both domain general.

In our paper, we show that the weighting of the channels at low frequencies is different for speech than vocals. To be more specific, we have added an analysis which directly compares those topographies by comparing the scaling factors at channels Fz and Pz (Figure 5g). At higher modulation frequencies (4-32 Hz), the difference between Pz and Fz scaling factors is significantly more positive for vocals than speech, albeit only weakly. But at lower modulation frequencies (0.5-4 Hz), the difference between Pz and Fz scaling factors is significantly more negative for vocals than speech because of the relatively larger increased weight of Fz to Pz in the vocals topography. Additionally, for vocals the Pz-Fz difference is significantly larger for the 250 ms model than the 2000 ms model, while for speech the 2000 ms shows larger difference than the 250 ms model, albeit more weakly. The effects for vocals show an interestingly larger difference because of the increased weighting over Fz at lower modulation frequencies, while speech appears to show the opposite.

Taken together with the specific- and general-model comparisons, we think the difference in reconstruction accuracy relative to the general model and the difference in Fz and Pz weightings indicate that the processing occurring at low frequencies is not domain-general. Still, it is possible that, because the modulation power at low frequencies for vocals is relatively smaller than speech, it may not be activating this low frequency mechanism, and thus we do not see the same domain general mechanism present for vocals. We have addressed this in the discussion:

“Still, there is some evidence that low-frequency tracking of speech is produced by a domain-general mechanism (Hamilton et al, 2018). A future study could determine if the low-frequency tracking we observed here is specific to speech by presenting both speech and single-instrument stimuli with phrase-level fluctuations in amplitude. Such a study could also clarify if the increased parietal weighting was not observed for vocals simply because there was not enough modulation power in their envelopes.” (l. 478-484)

*Minor:

* Introduction line 54: In the two papers cited (Oganian & Chang, 2019; Kojima et al., 2020), evoked responses are time-aligned to envelope edges which mark vowel-nucleus onsets, not syllable onsets. Although the two are highly correlated they have different theoretical implications.*

We have changed the sentence to read: “…respond particularly to envelope edges, which mark vowel-nucleus onsets and are highly correlated with syllable onsets in speech”. (l. 56-57)

** Figure 1B: Because "1/f" effects dominate the modulation spectrum, it's difficult to visually extract what the critical differences are between the four stimuli (aside from speech having a uniformly higher power). I suggest including a panel that plots normalized spectra for each music condition relative to speech (in the same vein as the reconstruction accuracies in figure 3E).*

We swapped the figures in Figure S1 (now Figure S1a) and Figure 1b. In this figure, the spectra were normalized to the average spectrum of the EEG. In effect, this gives an approximate signal-to-noise ratio if the brain tracked the envelope exactly and the background noise had a spectrum shaped like EEG. We made this a supplemental figure originally because we were concerned about the complexity of explaining it in the paper. We tried using log-linear detrending to remove the 1/f effects in the spectra, but this seemed to reduce the peak of the spectrum of speech at low frequencies, which we thought was especially relevant for our analysis and would be a disservice to the reader not to show it clearly. This has been described in lines 628-654 in the Methods.

** Lines 100-111: a general comment about the flow - at this point in the paper it is unclear how the narrowband modeling approach allows for a clear dissociation between the competing hypotheses. Only when stimulus general vs. specific models are compared can one infer anything about higher-order effects above and beyond what would be expected if neural differences just reflected acoustic ones. As the results currently read, this paragraph sounds like the narrowband modeling allows for such a dissociation.*

The last sentence of that paragraph stated our hypotheses, and we thought the reviewer was correct that it wasn’t clear how narrowband modeling would address the hypotheses. When we did this project, the analysis described here followed our broadband envelope reconstruction analysis, shown in Figure 7, where we found speech was reconstructed better. Here we wanted to address if those differences are a consequence of the frequency differences in envelope tracking across stimulus types.

We changed the way we phrased our hypothesis at the end that paragraph to make the motivation for using a narrowband analysis clearer: “This would allow us to identify if differences in envelope reconstruction that we observe using the broadband envelope (Figure 7, Methods) are due to differences in the modulation frequencies that are most strongly tracked. Alternatively, envelope tracking of speech could be better because of the relative variance in the modulations of the dB envelope.” (l. 116-120)

** The spline basis transform needs to be further motivated. What are the specific benefits of using such an approach above other methods? I am unfamiliar with this technique (and it’s safe to assume that most readers will also be). The transform seems to serve dual purposes of smoothing the PCs while also imposing a high frequency cut-off via the selection of knot sampling frequency. If these are the primary objectives, why not use more conventional smoothing and bandpass filtering techniques? There is mention of the need to control for temporal correlations but no explicit description of how splines may achieve this (or why temporal collinearity needs to be controlled if time-lags are included as features in the models).*

Our concern with implementing the bandpass filter as a preprocessing stage prior to modeling related to the ability to control reconstruction accuracies and model parameters. Even after bandpass filtering, because of spatial and temporal correlations in the EEG, a hyperparameter would need to be fit separately to each frequency range and potentially each stimulus type, which would complicate interpretability, especially of the model weights. Our use of principal components and splines to create the model using a common architecture across frequencies we thought was the best approach to allow us to compare reconstruction across frequencies and stimulus types.

We added a sentence in the paragraph where we first introduce the model design. Note that we have cut the reference to controlling spatial and temporal collinearities – While this was part of our intentions, it does not clarify why we did not use a bandpass filter step separately, as the reviewer highlighted, and we think this new version will be clearer:

“We chose to implement this filtering stage within the model itself, rather than as a separate stage of preprocessing, so that we could use an identical model architecture and common hyperparameters to investigate each frequency range (Figure 2a).” (l. 122-124)

** The collective effect of moving-average subtraction and spline transformation is a bandpass on the EEG data (figure 2C). However, on the stimulus end, only removal of the moving-average is applied. Theoretically this means that lower-frequency neural modulations could map onto higher-frequency envelope power? A brief explanation of why this is/isn’t the case is needed.*

The model could map onto envelope power, but not in its current design. By working with envelope power, it is assumed that the phase of the higher frequency is irrelevant. To quantify this, though, a nonlinear transformation of the dB envelope is required (for example, taking the absolute value of the analytic signal, as in the Hilbert transform). While it’s interesting, we think this is beyond the scope of the current study.

** Figure 5: Y-axis labels are missing from 5E-F (the values seem rather high to be the actual R^2 from correlating predicted vs. actual envelope, but perhaps this is because predictions are evaluated on a trial-by-trial basis?). More generally, the results shown in figure 5E-F are not immediately easy to square with the results of 5A. Given that the speech-specific model outperforms the general at low frequencies, why aren’t the general-to-specific model fits lower in this condition? If the scaling factor and circular shift is able to compensate for this difference, the results need to be visualized such that this becomes clear (e.g. show the unscaled and unshifted R^2 side-by-side with current data).*

The R-squared values shown in this plot are not related to the differences in reconstruction accuracy shown in 5A. Here we are comparing the model weights of the general model to the specific model by circularly shifting and scaling the model weights to best match the specific model on a trial-by-trial basis. The R-squared values are of the fits to the model weights, and they tend to be large (even sometimes for the null distribution) because we are optimizing the fits on a trial-by-trial basis.

However, the reviewer raises an interesting concern about why the R-squared fits aren’t lower for speech given that it outperforms the general model. For clarity, we have also included the R-squared values relative to the original general model weights as a supplemental figure (Figure S11). Without the scaling and shifting, the R-squared values are worse, and the values for speech are on par with those for orchestral and vocals. We show in Figure 5f that the shift is not significantly different than zero for speech. Thus, the improvement with scaling and shifting is produced by the effect of scaling. Since training the general model on music focuses the weighting on frontal channels (Figure 5c), we conclude from this that the topography difference in the scaling factors contributes to the difference in reconstruction accuracy between the speech-specific and stimulus general model at 0.5-4 Hz (Figure 5a).

** Relatedly, it's unclear why music-specific models (particularly rock) would exhibit differences from general models that actually hurt rather than improve performance across all frequencies. Authors should, at the least, include in the discussion some speculation as to why this may be (is it just a data limitation issue?).*

We think the effect that the reviewer refers to is mainly a data limitation issue. This can be somewhat clarified by looking at cross-stimulus models, which are trained on the same amount of data (see Reviewer #3’s comment and Figure S9). The model performance of the models trained and tested on the music stimuli are comparable to (and sometimes better than) the models trained on other stimulus types. Because the stimulus-general models contained more training data, it suggests that the issue the reviewer has highlighted is a data limitation issue. We have addressed this in the manuscript (see the response to Reviewer #3’s first comment).

*Miscellaneous:

* line 189 refers to figure 1b (not figure 2b).*

This now refers to Figure S1b (l. 222).

***Reviewer #3****: Zuk and co-workers present a thorough study on the envelope tracking of speech and music measured with EEG. The authors paid particular attention to potential biases on the reconstruction performance due to different stimulus types and thus added two additional processing steps to establish their envelope reconstruction models (PCA + spline basis). The results showed superior reconstruction of speech stimuli over music types (rock music, vocals extracted from rock pieces, classical music) that is presented to be unique to low frequencies and parietal channels.
I appreciate the extended analyses as well as the care that has been taken during control analyses and in interpreting the results. I also think that the study adds valid and important methodological points of discussion to the field of envelope tracking of speech and music.*

Thanks!

*However, I think that the main empirical finding(s) of this study could be better communicated. As is, the manuscript reads very analysis-heavy and undermines the impact of the empirical results. In addition, these main empirical findings require further tests.
Please find these and further suggestions in the main and minor points below.

Main points:

1. The main conclusion of the paper mentioned in the title and abstract is that speech is tracked ‘uniquely’ by low frequencies. These results mainly stem from section “Speech envelope tracking at low frequencies […]” (l.202-268) and are based on results presented in Figure 5, which aims to answer the question on whether model “differences affect neural tracking of the envelopes for the different stimuli” (l.219/220) and whether “speech tracking at low frequencies [is] a result of common processing across stimulus types” (l.223/224). This analysis compares the performance of the ‘specific’ model (training data are of the specific stimulus class) to the ‘general’ model (training data are trials across stimulus types) that is assumed to “capture consistent trends across all stimulus types relevant to envelope tracking” (l.227). This would hold only if each trial has a similar impact on models. However, as explained in this paper, music stimuli are more variable and thus might not affect models as much as compared to speech stimuli that show less variance. I suggest performing a cross-decoding/reconstruction analysis that would use a model of one stimulus type (e.g., speech) to reconstruct trials of the other stimulus types. Thus, the results would be similar to a confusion matrix where the specific models would lie on the diagonal. This way the questions above will be more directly answered and also the main conclusion of unique tracking of speech at low frequencies (Fig 5A) can be better examined.*

We thank the reviewer for suggesting this analysis. We ran this analysis, and we have included the resulting figure as a supplemental figure (Figure S9). The figure shows the median difference between the model trained on the same stimulus type as testing (what we call the “stimulus-specific model” in Figure 5) and a cross-stimulus model trained on one stimulus type and tested on another. Along the diagonal the differences are zero. Firstly, these results highlight our claim about the stimulus-general model performing better because of data limitation. In this plot, for the 250 ms model, the same-stimulus models for music do better than the cross-stimulus models. This is not the case for music at the lower frequencies. However, the speech-specific model notably outperforms the cross-stimulus models for both the 250 ms and 2000 ms models. This supports our original claim for neural tracking that is present for the speech stimuli but not the music stimuli.

We addressed this in the manuscript:

“To better understand these differences, we looked instead at the difference in performance between the stimulus-specific models and “cross-stimulus” model trained on one stimulus type but tested on another (Figure S9). We again found that the model trained and tested on speech did significantly better than the model trained and tested on other stimuli, with a larger difference in prediction accuracy for the 0.5-4 Hz model. Notably, we also found that the other music stimuli seemed to outperform the cross-stimulus models for the 4-32 Hz model, but not 0.5-4 Hz. This suggests that the worse performance compared to the stimulus-general model that we found in Figure 5a maybe have been partly an issue of limited data, since the stimulus-general model was trained on 24-28 trials compared to the 6-7 trials for the cross-stimulus models. However, the improvement in performance produced by the stimulus-general model is still smaller than the difference in performance between speech and the music reconstructions (for reference, see Figure S3 showing the reconstruction accuracy using non-normalized r-values). Thus, we do not think that increasing the amount of data would have improved the performance of music envelope reconstructions sufficiently to account for the difference with speech.” (l. 290-303)

*2. The other main conclusion that speech tracking was “uniquely associated with increased weighting of parietal channels” (l.202/3, also in abstract) is based on descriptive results presented in Fig 4d, lower plots. I do agree that these results are an indication but if this is to be a main finding, I suggest to statistically test whether the weighting of these channels is higher compared to the other stimulus types. If the statistics over model parameters are not straight-forward here, the permutations (null models) might provide a good normalization (similar to analysis of reconstruction accuracy (Fig 3D,E). Related to this, I wonder how this finding is interpreted.*

We have included an additional statistical analysis comparing Pz and Fz scaling factors (Figure 5g). Admittedly, the effects are weak for speech but they show the trends we discussed in the paper, with increased weighting over parietal channels for the 0.5-4 Hz model relative to the 4-32 Hz model. Interestingly, the opposite trend is observed vocals, showing increased weighting over frontal channels at lower frequencies. Together, it suggests that the processes for tracking speech and vocals at low frequencies are different, though we have further refrained from calling it “unique” tracking of speech given our limited dataset and other possible considerations.

We have included the following in the manuscript:

“To test these topographic differences statistically, we looked at the difference between parietal channel Pz and frontal channel Fz (Figure 5g). For the 4-32 Hz model, the difference between Pz and Fz was significantly larger for vocals than speech, although this effect was weak (Wilcoxon rank-sum test: z = 2.22, p = 0.027). For the 0.5-4 Hz model, this relationship flipped and speech showed a larger Pz-Fz difference (z = -4.20, p < 0.001). Comparing between frequency ranges, vocals showed a more negative Pz-Fz difference for low frequencies than high frequencies (Wilcoxon signed-rank test: z = 4.02, p < 0.001). Again, this relationship was flipped for speech, though the effects were weaker (z = -2.22, p = 0.026).” (l. 344-351)

*3. Please be clearer on the reason of using the unusual model using PCAs and a spline basis for envelope tracking. It is the main model used in this study and represents one major novelty. However, it is more complicated than previous envelope tracking approaches. For example, it would strengthen the paper to explain the advantages of 1) results of envelope tracking with different windows sizes and EEG filtering in comparison to the PCA-spline approach and 2) why it overcomes stimulus-specific acoustic regularities of the different stimulus types clearly in the main text. The latter is not clear to me and I do not follow how this analysis manages to abstract from inherent acoustics of stimuli affecting envelope tracking. Related to this point: In figure 2B, the ‘standard’ EEG envelope tracking provides higher reconstruction accuracy in comparison to all but one model type of the new approach and might make readers wonder about this tracking approach.*

We think the main benefit of the PCA-spline approach compared to EEG filtering is the ability to use a consistent model architecture for all frequency ranges, which makes comparisons across frequency and stimulus types easier. This concern is similar to Reviewer #2’s comment about our reasoning behind this approach, and we have added the following to the text:

“We chose to implement this filtering stage within the model itself, rather than as a separate stage of preprocessing, so that we could use and identical model architecture and common hyperparameters to investigate each frequency range (Figure 2a).” (l. 122-124)

The approach we use here is similar to other approaches that identify sets of hyperparameters to optimize the model (see Norman-Haignere et al, 2015; Norman-Haignere et al, 2019). In this sense, the hyperparameters we have chosen that optimize the model are based on the Natural Speech dataset, and only one hyperparameter pair should do best given that reconstruction accuracy is (on average) a concave function of the hyperparameter values (see Figure 2b). Given the success of ridge regression, we use this method for comparison. Combined, we expect the model’s optimum to match the standard approach. We thought it might do better than the standard approach, but this was not the case. We have included the following in the Methods to clarify this point for the reader: “Our method of identifying the optimal hyperparameters is similar to other modeling approaches that identify the hyperparameters using a cross-validation approach (Norman-Haignere et al, 2015; 2019).” (l. 755-757)

*Minor points:

1. It seems that the silence in sounds was not controlled. It’s important to check whether there are differences in this aspect between sound types. The examples in Fig 1A suggest for example that speech has longer pauses than other types. This could be an explanation of the superior tracking as for speech. As this cannot be changed, I suggest providing a statistic on silences/pauses (length and #occurences) for the different stimulus types. If differences are found, this should enter the discussion of the results.*

The reviewer is correct that silences could influence the results, and this is essentially our main point about low-frequency tracking, which captures the on-off fluctuations in the envelope. Quantifying those silences are tricky, though, because they exist during continuous speech and well as in long pauses between sentences. We tried quantifying these silences as the reviewer suggested, using -55 dB V as a threshold for silences in the speech, and we found that there were on average 379 silences of 0.13 duration. However, this is misleading because it fails to capture the variety of silences in the speech. Another issue was that the noise floor for the silences were higher for vocals than speech in some of the stimuli (this can be seen in Figure 1). In earlier versions we attempted to control for these silences, but the noise floor differences forced us to use different thresholds for the silences, and in the end we were concerned that it unfairly decreased the speech performance, especially since speech was obviously being tracked at the low frequencies where long silences are relevant. Overall, we think these silences are a relevant part of this low frequency tracking that naturally occur in speech, and we think those fluctuations are quite effectively represented by the low frequency power shown in our normalized spectra (Figure 1b).

*2. L. 236-240: I very much appreciate that potential contributions of eye movement on reconstruction accuracies are openly discussed and that the comparison of specific vs general model were reanalysed. However, as these did change the outcome of this analysis, I’m also wondering about the remaining results (I can imagine that speech but especially music might lead to movement locked to the envelope). Performing the same analyses after preprocessing dedicated to remove EEG associated with EOG or other non-brain sources could resolve this (see for example Zuk et al., 2020). Given that backward modelling of long, continuous sounds, EOG and other artifacts might not have a strong impact if blinks or movements are unrelated to the stimulus. Thus another way of showing that results do not depend on artifacts would be to examine whether eye movements occurred at specific times during the sound.*

We reran the envelope reconstruction analysis using 1) the eyeblink component for each subject, computed using fastICA and identified based on the topography and the presence of eyeblinks in the signal (as in Zuk et al, 2020), and 2) the peak times of the eyeblinks. We performed the second analysis because we realized that the topography of the eyeblink component overlaps with the spatiotemporal weights of the reconstruction model, especially at high modulation frequencies (see Figure 4), and the second analysis validated if the eyeblink times alone could be used for reconstruction. In (1) we found that reconstruction accuracies decreased for all stimulus types, but speech reconstruction especially was still significantly above chance (relative to zero using a Wilcoxon signed-rank test with Bonferroni correction, p < 0.001). When we did (2), none of the reconstructions were significantly above chance. These results have been referred to in the manuscript (l. 236-241), and the respective plots have been included in Figure S7.

*3. L.199-200. I do not follow this sentence. In contrast to what is stated, it was mentioned earlier that speech envelopes had lower cross-trial spectral variance (l. 190). I might miss a the differences here but it would be good to provide to which result of this section this suggestion is related to.*

Line 190 (in the previous draft of the manuscript) was considering an alternative hypothesis that speech reconstructions were better because the speech stimuli were more similar to each other on each trial. Generally, this would result in lower cross-trial spectral variance. We accidentally referred to a plot here that was cut from the manuscript, be we have chosen to include it again as a supplemental figure since our reasoning without it may be confusing (Figure S1b). The within-trial analysis referred to in this paragraph was meant to test this alternative hypothesis. We found that the speech reconstructions were still better, so we concluded that this could not explain our results, and instead differences in modulation SNR may play a role (l. 199-200 in the original manuscript). We think including the figure for cross-trial spectral variance should make these paragraphs clearer.

*4. l. 289: Figure 2b -> 1b?*

We think the reviewer may be referring to l. 189. This referred to a plot that was cut, but we have re-added it as Figure S1b.

*5. l.221 “speech tracking is better at low frequencies (0.5 – 4Hz) than music tracking […]”. Maybe I missed an important part but I understood that one main finding of the first section (Fig 3) was that speech envelopes are tracked better across all but the very lowest frequency range? I thus wonder why the emphasis was put on the 0.5-4Hz range (it was reported as the peak difference between speech and music stimuli, l.175 but all other bands were very similar).*

For convenience, we decided to focus on one low-frequency model that appeared to show the largest speech tracking relative to music, so we used the model with the peak difference (0.5-4 Hz). Similarly, this frequency range showed the largest improvement in the speech-specific model compared to the stimulus-general model (Figure 5a; note that 1-8 Hz fails to remain significant after removing “eyeblink subjects”). We examined models to a lowest frequency range of 0.0625-0.5 Hz thinking originally that was the limit of speech tracking, but when following up with the Natural Speech dataset we found that speech reconstructions were above chance with more data, suggesting also that speech may be weakly tracked in this range. Still, the reconstruction accuracies for Natural Speech were also largest on average at 1-8 Hz and 0.5-4 Hz, so we think this frequency range was a valid choice for examining low-frequency envelope tracking.

*6. Fig5E. to be consistent with 5F, indicating significances should be included*

Significance in Fig. 5e is indicated with the red asterisks above the dots in each stimulus type.

*7. L. 326-329. The fact that there are multiple sound sources (instruments) could lead to attention switches (between instruments and between single instruments and the aggregate music) and as such affect tracking that is shown to depend on attentional focus and state. The fact that the drums are tracked best and similarly well to the overall rock stimulus does not imply that paying attention to an isolated instrument would lead cannot approach speech tracking. As such, this might (partially) explain the superior speech tracking, which consisted of one source in isolation vs. a multi-source sounds for rock music.*

We considered the alternative hypothesis that subjects would be paying attention to individual instruments, but as the reviewer suggests, it does not match the envelope reconstruction accuracies for speech. These results are shown in Figure 6a. However, we do not think that if subjects were given a specific task of attending to the drums it would match the performance for speech because the vocals were also presented in isolation. We included a sentence in the discussion to highlight this fact, since we realized it was not specifically addressed: “Unlike the rock instruments, whose envelope reconstructions also did not match speech performance, the vocals stimuli were presented in isolation, suggesting that attention to an individual auditory object also may not explain the relatively high performance observed for speech.” (l. 458-467) Still, we think it would be a worth doing a follow up experiment to compare speech tracking with envelope tracking of instruments presented in isolation, as described in the discussion. (l. 480-486)

*8. Fig. 2B. Could you please provide an explanation (response letter suffices) on why the full model (128 PCs, no spline) performed worse than EEG model? Using all principal components should provide the same amount of information as the original EEG data. It might thus be due to the specific model type.*

This is an effect of overfitting. Note that no regularization was applied to these models when comparing to the “untransformed EEG” model, which used ridge regression. To make this clearer, we have added the following: “When a small number of principal components and splines are included, the model tends to underperform compared to models with more parameters (Figure 2b). Because no regularization was applied to the PCA & spline model, it also underperforms when all principal components are included.” (l. 155-158)

*9. Fig. 4C, D and Fig 5C: Please add information on how the intervals of the presented weight topographies were chosen (for example, according to weight time-courses (e.g., delay minimum weight +/-20ms)*

We chose these intervals so that they encompassed the peaks and troughs of the time-varying weights averaged across channels (Figure 4b). This is described in the legend of Figure 4c and d.

*10. l.43: don’t -> do not*

Done (l. 45)

*11. l.588: figure 2E does not exist, my guess is that this refers to Fig 1C.*

This was meant to refer to Figure 2c. We have changed the reference from 2e to 2c. (l. 782)
