# Supplementary material for: Envelope reconstruction of speech and music highlights stronger tracking of speech at low frequencies
Source: PLoS Comput Biol. 2021 Sep 17;17(9):e1009358. doi: 10.1371/journal.pcbi.1009358 (PMC8480853; doi:10.1371/journal.pcbi.1009358)
Supplement: Attachment — Submitted filename: NeuroMusic_reviewerresponses_2.docx [file pcbi.1009358.s016.docx]

**Envelope reconstruction of speech and music highlights stronger tracking of speech at low frequencies**

Responses to the reviewers

The line numbers listed refer to the version of the manuscript with tracked changes.

Dear Authors,

I have read your paper and your response to the reviewers. I have a few comments of my own. I appreciate the care you took to address the reviewers comments. Also you are trying to demonstrate something that is not easy. It is very hard for one to accept that speech is special when the other stimuli have such different modulation power (and clearly smaller as well shown in your figure 1B!) Much of your effect could just be do to SNR.. You work hard to show that it is more than that by examining the value of the weights and comparing goodness of fits to expected null values. One could argue that the best stimulus for the comparison would have match amplitude envelope spectrum but maybe lacking linguistic. But then one could also argue that such stimuli are not natural and, thus, not engaging the subjects.. In any case, I appreciate the effort you made by remain somewhat unconvinced about the effect size. The drumming control was clever and the small boost unexpected and clearly in favor of your argument. But maybe a rock musician involved in playing with the band could have EEG signals that are just as entrained...

We would like to thank the reviewer for their thoughtful comment on this point. We are very interested in how different types of musicianship (such as expert drummers) might change the reconstruction accuracies we observed here, but we think this is beyond the scope of this study.

I believe that your results speak for themselves and in the latest version you are indeed more careful in your statements. Here are some minor points that I would like you to address, maybe just by sending me response.

1. In terms of the novelty of your method: I am impressed by your overall rigor and I also appreciate that you are able to perform an analysis in octave bands. But its seems like you could have gotten the same results just by estimating the coherence and coherency (to look at systematic errors in phase) instead of r or R2 for your measure of goodness of fit - and it would give you the goodness of fit as a function of frequency after just fitting a regular regularized linear model. See for example (Hsu A, Borst A, Theunissen FE. Quantifying variability in neural responses and its application for the validation of model predictions. Network. 2004;15(2):91-109). Would the results be different?

Our approach of using PCA and splines instead of regularization was intended to allow for consistent comparisons across stimulus types and frequency ranges, since we use the same model architecture for all models. If we used regularization optimized to all stimulus types, it could have resulted in a model that was biased towards speech and suboptimal for music. If we used regularization to optimize to each stimulus type separately, it makes it more difficult to compare model weights across stimulus types, since the effect of regularization would likely vary across stimulus types. Hence, we decided to go with this alternative to regularization. This is described in more detail in the Methods section “Effects of frequency content on regularization-based modeling of envelope tracking.”

Besides this, though, we agree that coherence as a measure of reconstruction accuracy could have produced results similar to Pearson’s r. However, if this was used after a regularized model was optimized to the broadband envelope, coherence (as a function of frequency) could highlight the effect of regularization on shaping the envelope reconstruction rather than reflecting a measure of the frequencies involved in neural tracking (see Figure 7). Additionally, we previously worked with a measure of reconstruction accuracy as a function of frequency that was similar to coherence (we looked at the spectrum of the error in the reconstruction), but we found that the measure became very difficult to interpret at high frequencies where the signal power is low, and we were concerned that any form of smoothing might bias the results.

2. I would use the same yscale in fig 4A. Maybe -20 to 5 ? Also the very low band would show greater weights for Vocal than speech- no? It might be an interesting comparison

Below we have included the plot of Figure 4a but using the same y-axis for all stimulus types. We considered this when we were first writing the manuscript, but we noticed that this makes the variation in model weights for rock hard to see. Also, in Figure 4b we plot the weights for the 4-32 Hz and 0.5-4 Hz models of all stimulus types to make them easier to compare. Our main goal with Figure 4a is to show the trends in model weights for each stimulus type as the frequency content changes, and because of this we think it is more appropriate to keep the scales as is so that the weights of each stimulus type are more visible.

We also thought it was interesting that the vocals had such large model weights at low frequencies; this is also visible in Figure 4b, where the weights are comparable to speech. Still, it is notable that they do not contribute comparably to envelope reconstruction as they do to speech. We have added a sentence to make this point directly: “It is also notable that the magnitude of the vocals weights on average are comparable to speech, even though reconstruction accuracies are smaller at these frequencies.” (l. 255-257) We more rigorously compare the effectiveness of each model later with the stimulus-general model comparisons (Figure 5) and our follow up work contained in the Supplemental Information (Figure S9 and S10), which highlights that, despite the large model weights for vocals, it does not necessarily mean better reconstructions. As such, we also don’t think it is appropriate to compare them directly in Figure 4b either given our later analyses.

3. The fact that you get better reconstruction with the general model suggests that you are data limited and just more so for the stimuli other than speech almost certainly because it has a higher SNR. I think you address some of this in 5E by estimating the chance R2 distribution but I also think that it is hard to be completely convinced. I don't really know how to correct for differences in SNR when one is also using regularization. I know that you use the same hyper-parameters in your data analysis and so maybe it could just involve restricting the data set of the speech to match some form of integrated SNR? If you have addressed this, I did not completely follow your argument in the paper.

We note the possibility of a data limitation after examining cross-stimulus models in Figure S9. While the stimulus-general model was trained on more trials than the stimulus-specific models (since it was trained on multiple stimulus types), the cross-stimulus models were trained on similar numbers of trials. We find that at 4-32 Hz the original models trained on music do better than the cross-stimulus models. If this is compared to Figure 5a, it suggests that the poor performance of the music-specific models compared to the stimulus-general models (Figure 5a) is based on a data limitation. We noted this in the results section, but we have tried to make this point clearer (see below). We also note that the r value improvement of the stimulus-general model is still well below the r values for speech (Figure S3), so the difference in speech and music envelope reconstructions cannot be entirely explained by limited data. Unfortunately, we do not have enough EEG data during music listening to analyze this further.

“Notably, we also found that the other same-stimulus models trained and tested on music stimuli seemed to outperform the cross-stimulus models for 4-32 Hz, but not 0.5-4 Hz. This was different from our finding in Figure 5a, where the stimulus-specific models trained on music performed worse than the stimulus-general model. We think this indicates an issue of limited data, since the stimulus-general model was trained on 24-28 trials compared to the 6-7 trials for the cross-stimulus models. However, the improvement in performance produced by the stimulus-general model is still smaller than the difference in performance between speech and the music reconstructions (for reference, see Figure S3 showing the reconstruction accuracy using non-normalized r values). Thus, we do not think that increasing the amount of data would have improved the performance of music envelope reconstructions sufficiently to account for the difference with speech.” (l. 284-294)

Also, R^2^ used in Figure 5e refers to the fit between the stimulus-specific and stimulus-general model weights after scaling and circularly-shifting the stimulus-general model (Figure 5d). This is unrelated to the r values shown for reconstruction accuracy. Since this was a source of confusion in the previous round of reviews, we have added a sentence to the results to clarify this point.

“We used R^2^ to quantify the fit of the scaled and shifted stimulus-general model to the stimulus-specific model (note that this is different than Pearson’s r, which we use as a measure of envelope reconstruction accuracy).” (l. 314-316)
